# Health-Related Physical Fitness and Quality of Life in Children and Adolescents With Isolated Left-to-Right Shunt

**DOI:** 10.3389/fped.2019.00488

**Published:** 2019-11-22

**Authors:** Angeles Fuertes Moure, Michael Meyer, Anna-Luisa Häcker, Barbara Reiner, Leon Brudy, Renate Oberhoffer, Peter Ewert, Jan Müller

**Affiliations:** ^1^Unit Pediatric Cardiology, Hospital Materno-Infantil Teresa Herrera, Coruna, Spain; ^2^Department of Pediatric Cardiology and Congenital Heart Disease, German Heart Centre Munich, Technical University Munich, Munich, Germany; ^3^Institute of Preventive Pediatrics, Technical University Munich, Munich, Germany

**Keywords:** simple severity, functional outcome, congenital heart disease, shunt leison, motor competence, quality of life

## Abstract

**Objective:** Atrial (ASD) and ventricular septal defects (VSD) represent the most common congenital heart defects (CHD) and are considered simple and curable. This study investigates long-term functional outcomes in children with such defects.

**Patients and Methods :** We examined 147 patients (74 girls, 12.1 ± 3.5 years) with isolated shunts (ASD: 54%, VSD: 46%) for their Health-Related Physical Fitness (HRPF) and Health-Related Quality of Life (HRQoL). Native condition was present in 58 patients, interventional closure of the defect was performed in 42 and surgical closure in 47. For comparison, a healthy control group (CG) of 1,724 children (48.9% girls, 12.8 ± 2.8 years) was recruited within two recent school projects.

**Results:** After adjustment for age and sex, children with ASD and VSD presented lower HRPF (z-score healthy peers: 0.02 ± 0.73, ASD: −0.41 ± 0.73, *p* < 0.001; VSD: −0.61 ± 0.73, *p* < 0.001) then healthy peers. Transferred into percentiles, VSD were on the 26th and ASD on the 34th percentile of the healthy peers. HRQoL did not differ between peers and CHD with isolated shunts (healthy peers: 76.1 ± 9.7, ASD: 76.2 ± 9.9, *p* = 0.999; VSD: 78.7 ± 9.7, *p* = 0.316). Regarding the surgical history of the shunts (native, percutaneously treated, surgically treated), there were also no difference in-between these three states, nor differed HRPF and HRQoL in-between gender.

**Conclusions:** Children with ASD or VSD have impaired HRPF but normal HRQoL. Early childhood sports promotion could be a good measure to counteract these restrictions in HRPF at an early stage.

## Introduction

Ventricular (VSD) and atrial septal defects (ASD) represent the two most common congenital heart diseases (CHD), are considered as simple CHD, and constitute over 60% of the total CHD population (1, 2). Some of these defects that are hemodynamically not relevant and do not require surgery or intervention. The others are considered after successful intervention or operation as surgically cured. Nevertheless, they represent a chronic diseased population and even those simple and often untreated defects have increased long-term mortality and cardiac morbidity (1).

The current change in the landscape of pediatric clinical management of patients with CHD is influenced by the increasing number and life expectancy of adults with CHD (3). This approach focuses not only on quantitative results related to mortality, re-interventions and hospitalization, but also on functional aspects such as health-related quality of life (HRQoL), physical activity and fitness, as well as the prevention of chronic diseases. Within the field of CHD, more attention in terms of research is given to the more complex lesions. But even in the simple CHD, VSD, and ASD in particular, functional limitations in terms of HRQoL (4, 5), motor behavior or health-related physical fitness (HRPF) (6, 7) as well as exercise capacity exist (8). Paradoxically at least in HRQoL, limitations are often as detrimental as in complex lesions (4, 5). Those imitations could affect the physical, social, emotional, and cultural development that worsen HRQoL and activity behavior in the long-term (9–11). The latter is crucial as physical activity is a cornerstone in cardiovascular prevention. In light of the above, the objective of this study was to investigate children with ASD and VSD in comparison to a healthy peer group.

## Patients and Methods

### Study Subjects

From May 2014 to March 2019 we examined 1,203 children 6–18 years with various CHD from the outpatient department of the German Heart Center Munich during their routine follow-up visit for their HRPF and HRQoL. The patients were asked for participation when they registered in our outpatient department and then directly enrolled into the study.

Of those enrolled, 147 patients (74 girls, 12.1 ± 3.5 yeas) have isolated shunts, which 80 patients (54%) had an ASD and 67 patients (46%) a VSD. All study subjects were non-syndromic and did not have any restrictions to exercise and those surgically or percutaneously treated had no signs of shunt residuals or valve regurgitation. All patients were in NYHA 1 or 2.

**Figure 1:**
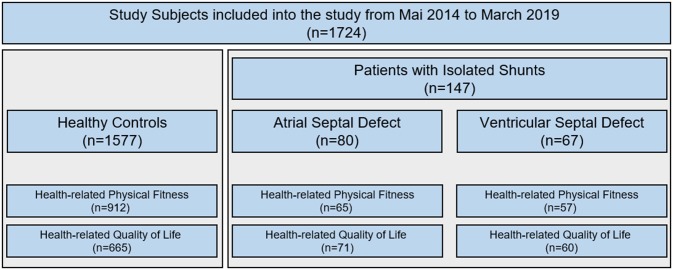
Patients inclusion.

Not all patients could perform both procedures, the fitness test (HRPF) and the questionnaire (HRQoL). Thus, from the 147 patients a subset of 122 patients performed the HRPF test and 131 filled out the HRQoL questionnaire (Figure 1). This did not happen systematically and should therefore not have a sampling bias on the results. All included patients have at least one measure of HRPF or HRQoL. The study characteristics are displayed in Table 1. Native condition were still present in 58 patients, interventional closure of the defect in the catheter laboratory have been performed in 42 and surgical closure of the shunt was performed in 47.

**Table 1:** Study characteristics.

	**Healthy controls** **(*n* = 1724)**	**Patients with isolated shunts** **(*n* = 147)**	*p*-value^*^	**Atrial septal** **defect** **(*n* = 80)**	**Ventricular septal defect** **(*n* = 67)**	*p*-value^*^
Sex (female)	978 (48.9%)	74 (50.3%)	0.776	45 (56.3%)	29 (43.3%)	0.137
Age (years)	12.8 ± 2.8	12.1 ± 3.5	**0.001**	11.5 ± 3.6	12.8 ± 3.4	**0.026**
Body weight (kg)	47.0 ± 14.2	43.9 ± 17.7	**<0.003**	42.0 ± 18.0	45.9 ± 17.1	0.206
Body length (cm)	156.0 ± 13.9	149.9 ± 19.9	**<0.001**	146.3 ± 20.2	154.2 ± 18.8	**0.016**
Body mass index (z-score)	0.04 ± 1.04	−0.12 ± 1.03	0.079	−0.02 ± 1.26	−0.2 ± 0.96	0.266

* *Chi-square test or T-test for independent samples, significant values (p < 0.050) are presented in bold*.

The healthy control group (CG) was recruited within two recent projects from this decade “Star moments of health (Sternstunden der Gesundheit)” (12) and “Catch the Pulse Wave” (13) that aimed on general physical, psychological and vascular health of children in Bavaria and followed the same standard operating procedure (Table 1 and Figure 1).

This study was carried out in accordance with the recommendations of recent guidelines with written informed consent from all children and their guardians in accordance with the Declaration of Helsinki. The protocol was approved by the ethical committee of the Technical University of Munich (project number: 314/14) and is part of the FOOTLOOSE (Functional outcome in children and adolescents with congenital heart disease) project (Deutsches Register Klinische Studien: DRKS00018853). Small parts of the data have recently been published elsewhere (5, 14, 15). In detail, this concerns only the results of HRQoL in 30 patients with ASD and 32 patients with VSD (5).

### Health-Related Physical Fitness (HRPF)

As described previously (16) HRPF was tested by five tasks of the FITNESSGRAM® test battery in standardized order. In brief, it's a fitness test where curl-ups and push-ups were performed to test upper body strength. Upper body flexibility was tested by shoulder stretch task and lower body flexibility by the sit and reach test. For trunk extensor strength and flexibility, the trunk lift (distance chin to the ground after lifting the upper body out of the prone position) was applied for two tries (17, 18). Detailed insights in the testing procedure can be accesses from the online supplement of our previous study in TCPC patients (18). For the analyses the mean of the left and right-sided test in shoulder stretch and sit and reach were used as well as the maximum out of two trunk lifts. The raw values from the FITNESSGRAM® were transformed into standard deviation scores (z-score). The tests were carried out by one person out of three experienced and well trained examiners.

### Health-Related Quality of Life (HRQoL)

For the assessment of HRQoL from a subjective perspective we used the paper version of the KINDL®, an age-adapted, self-report questionnaire that is internationally accepted and standardized (19, 20). The children had to fill in the questionnaire without help, only a few of the younger children, who still had problems with reading, had individual questions read aloud. As previously described (5) the questionnaire consists of 24 items (referring to the past week) that have to be answered on a 5-point Likert scale (never, seldom, sometimes, often, and always). Those items were then converted into a HRQoL score and into six subscales (physical, emotional well-being, self-esteem, family, friends, everyday functioning) which has a range from 0 (worst) to 100 (best). The questionnaire can be accessed via this URL https://www.kindl.org.

### Data Analyses

Descriptive data was expressed in mean values and standard deviation (mean ± SD).

Simple group comparison between patients with isolated shunt and healthy CG, and the comparison of data from ASD and VSD were analyzed with Student's *t*-test for independent samples or chi square test if appropriate.

The raw values from the FITNESSGRAM®were transformed into standard deviation scores (z-score) based on the test results of the CG. First, LMS values for the CG were calculated according to Cole (21) using R-Studio (version 0.99.879, R-Studio Inc.) with the module extensions *gamlss* (version 3.4-8) and AGD (version 0.34). Second, these LMS values were used to calculate z-score values for the ASD and VSD patients.

For comparison of HRPF and HRQoL within the CG and children with ASD and/or VSD, a generalized linear model (GLM) was used to assess mean differences between groups. For the comparison of HRPF a simple GLM without covariates was used since LMS transformation into z-scores already adjusted for sex and age.

All calculations were performed using SPSS 23.0 software (IBM Corp., Armonk, NY, USA). Two-sided *p* < 0.050 were considered significant.

## Results

As Table 1 illustrates, children with isolated shunts were slightly younger, smaller and lighter as the healthy controls and also some other small differences between children with ASD and VSD occurred.

After adjustment for age and sex (Table 2), children with ASD (z-score: −0.41; *p* < 0.001) and VSD (z-score: −0.61; *p* < 0.001) presented lower HRPF compared to the healthy CG.

**Table 2:** Comparison of patients with ASD and VSD to healthy controls with regard to health-related physical fitness and health-related quality of life.

	**ASD**	**Mean difference** **CG to ASD**	**Healthy control group (CG)**	**Mean difference** **CG to VSD**	**VSD**
**Health-related physical fitness**	***n*** **=** **65**		***n*** **=** **912**		***n*** **=** **57**
Motoric score (z-score)	−0.41 ± 0.73	**−0.43 (*****p*** **<** **0.001)**	0.02 ± 0.73	–**0.63 (*****p*** **<** **0.001)**	−0.61 ± 0.73
Curl-ups	−0.21 ± 1.09	−0.25 (*p* = 0.227)	0.03 ± 1.09	−0.18 (*p* = 0.679)	−0.14 ± 1.09
Push-ups	0.18 ± 1.05	0.16 (*p* = 0.695)	0.02 ± 1.05	0.12 (*p* = 0.999)	0.14 ± 1.05
Shoulder stretch	−0.52 ± 1.19	**−0.53 (*****p*** **=** **0.002)**	0.01 ± 1.19	**−0.74 (*****p*** **<** **0.001)**	−0.73 ± 1.19
Sit and reach	−0.50 ± 1.21	**−0.49 (*****p*** **<** **0.001)**	−0.01 ± 1.21	**−0.98 (*****p*** **<** **0.001)**	−0.99 ± 1.21
Trunk lift	−0.96 ± 1.30	**−0.96 (*****p*** **<** **0.001)**	−0.00 ± 1.30	**−1.35 (*****p*** **<** **0.001)**	−1.35 ± 1.30
**Health-related quality of life**	***n*** **=** **70**		***n*** **=** **665**		***n*** **=** **60**
Total score in KINDL (score)	76.2 ± 9.9	0.1 (*p* = 00.999)	76.1 ± 9.7	2.6 (*p* = 0.316)	78.7 ± 9.7
Physical well-being	71.2 ± 15.1	–**4.6 (*****p*** **=** **0.039)**	75.9 ± 14.8	3.1 (*p* = 0.330)	79.0 ± 14.8
Emotional well-being	80.0 ± 11.9	−3.0 (*p* = 0.123)	83.0 ± 11.6	0.2 (*p* = 0.999)	83.2 ± 11.6
Self-esteem	70.7 ± 17.5	4.1 (*p* = 0.177)	66.6 ± 17.2	2.3 (*p* = 0.953)	68.8 ± 17.1
Family	83.8 ± 14.8	−1.0 (*p* = 0.999)	84.8 ± 14.4	0.5 (*p* = 0.999)	85.3 ± 14.4
Friends	78.8 ± 14.4	0.8 (*p* = 0.999)	78.0 ± 14.1	**4.9 (*****p*** **=** **0.032)**	82.9 ± 14.1
Everyday functioning	72.4 ± 16.4	3.8 (*p* = 0.222)	68.7 ± 16.1	3.0 (*p* = 0.480)	71.7 ± 16.2

*ASD, atrial septal defect; VSD, ventricular septal defect; CG, control group. Significant values (p < 0.050) are presented in bold*.

**Figure 2:**
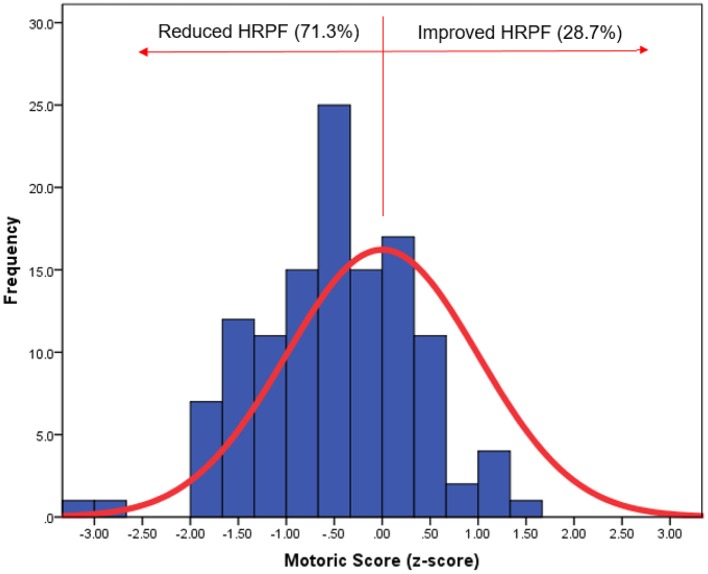
Distribution of health-related physical fitness of patients with isolated shunts (blue) according to the almost normal distribution of healthy controls (red line). HRPF, health-related physical fitness.

Compared to their healthy peers, patients in the VSD group were on the 26th percentile while those in the ASD group were on the 34th percentile (Figure 2). By analyzing the five tasks of the FITNESSGRAM® the main limitations came from the mobility and flexibility tasks, shoulder stretch, sit and reach and trunk lift (Table 2).

HRQoL did not differ between peers and CHD with isolated shunts (healthy peers: 76.1 ± 9.7, ASD: 76.2 ± 9.9, *p* = 0.999; VSD: 78.7 ± 9.7, *p* = 0.316). There were only two differences in the subscales (Table 2).

Patient characteristics were also stratified by native conditions, percutaneously treated lesion and surgically treated lesions (Table 3) and compared with their HRPF and HRQoL to the healthy control group (Table 4). All three groups showed significant limitations in HRPF in comparison to the healthy control group (all *p* < 0.01) and patients percutaneously treated showed higher HRQoL (*p* = 0.043). There were no differences in-between the patients with native conditions, percutaneously treated lesion and surgically treated lesions in terms of HRPF and HRQoL.

**Table 3:** Patient characteristics divided by native conditions, percutaneously treated lesion and surgically treated lesions.

	**Native conditions** **(*n* = 58)**	**Percutaneously treated lesion** **(*n* = 42)**	**Surgically treated** **(*n* = 47)**	*p*-value^*^
Sex (female)	26 (45.6%)	25 (59.5%)	23 (49.9%)	0.340
Age (years)	11.6 ± 3.4	11.2 ± 3.7	13.5 ± 3.2	**0.002**
Body weight (kg)	41.4 ± 16.3	41.1 ± 19.2	49.6 ± 16.8	**0.029**
Body length (cm)	148.1 ± 18.2	144.1 ± 21.3	157.2 ± 18.8	**0.005**
Body mass index (z-score)	−0.19 ± 1.17	−0.12 ± 1.38	−0.03 ± 0.96	0.798
Systolic BP (mmHg)	112.6 ± 11.8	112.6 ± 10.4	114.4 ± 9.1	0.672
Diastolic BP (mmHg)	65.4 ± 7.4	64.0 ± 7.4	65.3 ± 6.6	0.658

*BP, blood pressure*.

* *Chi-Square test, T-test for independent samples, significant values (p < 0.050) are presented in bold*.

**Table 4:** Comparison of patients with isolated shunts to healthy controls with regard to health-related physical fitness and health-related quality of life.

	**Healthy control group**	**Native conditions**	**Percutaneously treated lesion**	**Surgically treated**
**Health-related physical fitness**	***n*** **= 912**	***n*** **= 46**	***n*** **= 32**	***n*** **= 44**
Motoric score (z-score)	0.02 ± 0.73	−0.41 ± 0.73 **(*****p*** **= 0.001)**	−0.53 ± 0.74 **(*****p*** **< 0.001)**	−0.59 ± 0.74 **(*****p*** **< 0.001)**
**Health-related quality of life**	***n*** **= 665**	***n*** **= 54**	***n*** **= 37**	***n*** **= 39**
Total score in KINDL (score)	76.1 ± 9.7	78.5 ± 9.8 (*p* = 0.407)	80.4 ± 9.9 **(*****p*** **= 0.043)**	77.4 ± 9.9 (*p* = 0.999)

*Significant values (p < 0.050) comparing patients with isolated shunts to the healthy control group are presented in bold*.

There were also no gender differences in HRPF motor score and total HRQoL score.

## Discussion

The present study showed that children with ASD and VSD present significant lowered HRPF when compared with their healthy peers, regardless of the type of defect or the surgical history. However, their HRQoL is unaffected and normal. Those findings were more or less similar in ASD and VSD without significant differences between the groups.

### Health-Related Physical Fitness (HRPF)

In patients with CHD much attention is given to exercise capacity and recently, another study (22) has confirmed the persistent limitations in peak oxygen uptake in children with CHD. However, HRPF does not only refer to simple cardiovascular endurance, but also to muscular strength, flexibility and motor skills (16). Comprehensive evaluation is therefore important since e.g., muscular strength and balance are essential components of motor skills and vice versa. All these components are crucial determinants of exercise capacity in addition to heart function alone and significant for different tasks of daily life.

In terms of HRPF, muscle strength and motor development, studies (6, 23) report limitations and those impairments were seen even in those patients with non-existent or mild sequelae like in patients ASD and VSD. Overprotection, reluctant physicians to encourage physical activity, and a lack of recommendations for parents and teachers are social factors that are frequently discussed in this context (24). Indeed, Fricke et al. (7) had shown lower time-related recruitment of different muscular fibers (intra- and intermuscular coordination) in children with CHD which can be a result of less movement experiences due to a hypoactive lifestyle. Fewer muscular recruitment subsequently leads to reduced muscular power. However, that was not the case in our study as push and curl-ups were similar compared to healthy children. This is an encouraging result as generalized muscle weakness is associated with reduced lung function and reduced exercise capacity in adults (25, 26). This fact causes a current paper to speculate about strength training in CHD to boost respiratory function (27).

In terms of limited shoulder, hamstrings, trunk mobility, and strength a possible explanation is cardiothoracic surgery. After open heart surgeries the healing process features fibrotic tissue proliferation and remaining surgical scars. Both limits thoracic mobility which has shown to be associated with reduced lung and exercise capacity (28). Finally, preoperative cyanosis, circulatory arrest, and postoperative factors had shown to lead to neurological limitations and it is likely that they also influence HRPF (23, 29). However, since we could not observe differences in regard of HRPF depending on the surgical history of the shunts (native, interventional closure, surgical closure) it is more likely that hypoactive lifestyle is the reason for the present limitations. It could be assumed that all of those factors contribute to some extend to the limited trunk, shoulder, hamstrings mobility measured in our study. In order to avoid this situation of deconditioning, cardiac rehabilitation programs should be mandatory after surgery. Those are greatly underutilized although they have shown that they are effective (9, 30, 31). However, questions remain regarding the optimal structure and efficacy of the programs, but research is on. In addition to rehab, doctors should promote sport from early childhood, offering comprehensive individualized advice and education for which there are currently numerous recommendations from experts (32, 33), but instead they often hold back on clear encouragement to be active. Also sociocultural barriers, such as the media, parents, and schools, influence the physical activity experiences of pediatric heart patients and contribute toward low participation (34).

### Health-Related Quality of Life (HRQoL)

Although there are many publications on HRQoL in children and adolescents with CHD (4, 5), specific studies in patients with isolated shunt are missing. As in our previous report (5) on children and adolescents with different CHD, HRQoL of children with ASD and VSD in this study were at least as high as in healthy counterparts. This is in good agreement with other studies that examined HRQoL in children with simple CHD such as Hövels-Gürich et al. who tested 20 children with VSD (35). Laane et al. found also no reduced HRQoL in neonatal diagnosis of a later spontaneously closed ventricular septal defect (36). In patients with ASD, unfortunately specific data is available just in adults, but these also report on excellent HRQoL after ASD closure (37). Nevertheless, precise and more specific research in single CHD subgroups is necessary. Here the different subdomains of HRQoL should be considered, because there was no clear trend in our study and differences are probably just statistical artifacts. It would be important to know here exactly whether these patients in the school, emotional, or physical area judge themselves better or worse than healthy peers to tailor psychological support.

## Limitations

Unfortunately, we cannot give exact numbers on participation rate because we have to approach and include the patients on site, depending on the feasibility of the department's daily routine. Also due to the busy department's daily routine, to time constraints of patients and medical doctors in the outpatient clinic, it was not possible to perform all test procedures in all patients completely (Figure 1). This did not happen systematically and should therefore not have a sampling bias on the results.

This study refers to patients in regular aftercare in a tertiary center that recommends physical activity very liberally which represents a possible sampling bias. Unfortunately, the study lacked additional clinical and functional parameters, like measures from cardiopulmonary exercise testing, oxygen saturation, and hemodynamic measures derived from ultrasound or cardiac magnetic resonance. Also socio-economic status, a proven confounder of HRQoL, was not assessed. Due to time constraints in our outpatient department it was not possible to collect all data all children with ASD or VSD. However, this did not happen systematically and therefore no sampling bias should exist. Finally, since three different person conducted the fitness test there might be a possible bias. However, all examiners were experienced in testing and have regularly trained and updated each other in the execution of the test.

## Conclusion

Children with ASD or VSD have impaired HRPF mainly due to limitations in mobility and flexibility tasks, but have normal HRQoL. Early childhood sports promotion could be a good measure to counteract these restrictions in HRPF at an early stage.

## Data Availability Statement

The datasets generated for this study are available on request to the corresponding author.

## Ethics Statement

This study was carried out in accordance with the recommendations of recent guidelines with written informed consent from all subjects. All subjects gave written informed consent in accordance with the Declaration of Helsinki. The protocol was approved by the ethical committee of the Technical University of Munich (project number: 314/14).

## Author Contributions

AF analyzed the data and drafted the manuscript. MM, A-LH, BR, and LB sampled the data and gave important input for revising the manuscript. RO and PE were responsible for the conception and design of the study and gave important input for revising the manuscript. JM was responsible for the conception, design of the study, sampled parts of the analyzed the data, and drafted the manuscript.

### Conflict of Interest

The authors declare that the research was conducted in the absence of any commercial or financial relationships that could be construed as a potential conflict of interest.
